# Sweet taste perception not altered after acute sleep deprivation in healthy young men

**DOI:** 10.1007/s11818-013-0606-0

**Published:** 2013-05-30

**Authors:** P.S. Hogenkamp, E. Nilsson, C.D. Chapman, J. Cedernaes, H. Vogel, S.L. Dickson, J-E Broman, H.B. Schiöth, C. Benedict

**Affiliations:** 1 Department of Neuroscience, Uppsala University, 751 24 Uppsala, Sweden; 2 Department of Physiology/Endocrinology, The Sahlgrenska Academy at the University of Gothenburg, 405 30 Gothenburg, Sweden

**Keywords:** Sleep loss, Satiety, Food intake, Preload, Ghrelin, Schlafmangel, Sättigung, Nahrungsaufnahme, Vorspannung, Ghrelin

## Abstract

**Background:**

We hypothesized that acutely sleep-deprived participants would rate ascending concentrations of sucrose as more intense and pleasant, than they would do after one night of normal sleep. Such a finding would offer a potential mechanism through which acute sleep loss could promote overeating in humans.

**Method:**

A total of 16 healthy normal-weight men participated in 2 conditions: sleep (permitted between 22:30 and 06:30 h) and total sleep deprivation (TSD) respectively. On the morning after regular sleep and TSD, circulating concentrations of ghrelin and glucose were measured. In addition, participants hunger level was assessed by means of visual analogue scales, both before and after a caloric preload. Finally, following the preload, participants rated both intensity and pleasantness of six orally presented yogurt probes with varying sucrose concentrations (2–29 %).

**Results:**

Feelings of hunger were significantly more intense under both fasted and sated conditions when subjects were sleep-deprived. In contrast, the change in hunger induced by the preload was similar between the sleep and TSD conditions. Plasma concentrations of ghrelin were significantly higher under conditions of TSD, whereas plasma glucose did not differ between the conditions. No effects were found either on sweet taste intensity or on pleasantness after TSD.

**Conclusion:**

One night of TSD increases morning plasma concentrations of the hunger-promoting hormone ghrelin in healthy young men. In contrast, sweet taste perception was not affected by nocturnal wakefulness. This suggests that an altered sweet taste perception is an unlikely mechanism by which TSD enhances food intake.

## Abstract (de)

**Hintergrund:**

In dieser Studie untersuchten wir, ob normalgewichtige Probanden, die in der Nacht vor dem Test nicht geschlafen hatten, Joghurtproben mit unterschiedlichem Süßegrad als geschmacklich angenehmer und intensiver wahrnahmen. Ein solcher Befund könnte erklären, warum Menschen nach akutem Schlafentzug dazu neigen, mehr zu essen.

**Methode:**

Sechzehn gesunde normalgewichtige Männer nahmen unter 2 Bedingungen teil. In der einen Sitzung durften die Probanden zwischen 22:30 und 6:30 Uhr schlafen. In der anderen experimentellen Sitzung wurden die Versuchsteilnehmer die ganze Nacht wachgehalten. In beiden Sitzungen wurde dann am nächsten Morgen um 7:30 Uhr Blut zur Bestimmung von Ghrelin und Glukose abgenommen. Darüber hinaus gaben die Probanden ihren Hunger auf einer Skala von 0 (kein Hunger) bis 100 (sehr hungrig) vor und nach dem Verzehr einer Testmahlzeit an. Abschließend probierten die Versuchsteilnehmer in zufälliger Reihenfolge 6 Joghurtproben mit unterschiedlichem Süßegrad (2–29 %). Mithilfe von Skalen schätzten die Probanden den persönlichen Genusswert und die Süße für jede einzelne dieser Proben ein.

**Ergebnisse:**

Am Morgen nach der durchwachten Nacht waren die Probanden deutlich hungriger, sowohl vor als auch nach Verzehr der Testmahlzeit. Außerdem wiesen die Probanden erhöhte Konzentrationen des Hungerhormons Ghrelin im Blut auf. Im Gegensatz dazu gab es weder signifikante Unterschiede in der Blutglukose noch in der Wahrnehmung des Süßgeschmacks zwischen der Schlaf- und Schlafentzugsbedingung.

**Fazit:**

Unsere Ergebnisse deuten an, dass die erhöhte Nahrungszufuhr nach Schlafentzug, die bei jungen erwachsenen Menschen in anderen Studien nachgewiesen werden konnte, eher durch eine veränderte Produktion von Appetithormonen, wie z. B. die des Ghrelins, zurückzuführen ist und weniger durch Veränderungen der Süßwahrnehmung hervorgerufen wird.

Short sleep duration is considered a risk factor for weight gain in both children and adults [1]. A breadth of recent evidence suggests that this relation is causal. For instance, experimental sleep loss in healthy young men has been linked to reduced physical activity [2], increased appetite for sweet foods [3, 4], increased circulating ghrelin levels [4, 5], and increased neural responses to images of palatable foods [6]. This latter observation warrants further attention: when viewing food images, sleep-deprived men show enhanced neural activation in brain regions involved in both gustatory and reward processing, including the nucleus accumbens, thalamus, insula, and anterior cingulate cortex. This, in conjunction with previous observations that sleep-deprived men show an increased appetite for sweet foods [3], led us hypothesize that one night of total sleep deprivation (TSD) would enhance perception of sweet taste in healthy young men. To this aim, sweet taste perception was tested in 16 male subjects after either a night of normal sleep or TSD. Previous studies have shown that TSD affects both plasma ghrelin and glucose concentrations [4, 7]. Ghrelin is mainly produced by the stomach and stimulates food intake [8]. In addition, ghrelin has previously been linked to taste perception [9, 10] and food reward evaluation [11, 12] in animal studies. Thus, plasma levels of total ghrelin and glucose, as well as subjective hunger feelings were measured after sleep and TSD respectively.

## Methods

### Participants

A total of 16 healthy male subjects participated in the experiments (age 23 ± 0.9 years; body mass index 23.6 ± 0.6 kg/m^2^; all non-smokers, with a self-reported regular sleep–wake rhythm [i.e., ~8 h sleep/night] during the 6 weeks before the experiments, and not on any medication). Sleep disturbances during the intervention were excluded by electroencephalography (EEG) sleep monitoring. The design included an adaptation day including an overnight sleep that served to habituate participants to the experimental setting. The study was approved by the Regional Ethical Review Board in Uppsala, and the procedures followed were in accordance with the Helsinki Declaration. All participants gave written informed consent and were paid for their participation in the study.

### Study design and procedure

In a randomized and balanced crossover design, each subject participated in two conditions: 8-hours of sleep opportunity (‘sleep’) and total sleep deprivation (TSD). Upon arrival, participants started their adaptation day, which comprised a night of sleep followed by a standardized day in which they received three regular meals and one snack in fixed amounts, and two 30-min walks. This day was followed by a nighttime intervention period (22:30–06:30 h) in which subjects slept or stayed awake, after which they conducted the sweet intensity ratings as described below. Blood was sampled at 07:30 h after the nighttime intervention day. In the sleep condition, lights were turned off at 22:30 h, and switched on the next morning at 06:30 h. Polysomnography was performed using Embla A10 recorders (Flaga hf, Reykjavik, Iceland) and comprised EEG, electrooculography (EOG), and electromyography (EMG). Sleep stages were determined according to standard criteria [13] by an experienced scorer blinded to the study hypothesis. To keep subjects awake in the TSD condition, they were allowed to spend their time with a selection of movies, games, and books, they had access to bottled water, and lights were kept on. Participants were continuously monitored by the experimenters.

In the morning after regular sleep or nocturnal wakefulness, subjects were requested to rate their appetite sensations (hunger, fullness, desire to eat, prospective consumption) and thirst on a 100-mm visual analog scale (VAS) at 07:00 h. A blood sample was taken at 07:30 h for hormonal measurements, after which participants again rated their appetite sensations at 08:00 h. To minimize the potential confound of hunger evoked by TSD, immediately after these ratings, they received a caloric preload consisting of 2 packages (500 ml in total; 100 kcal/100 g) of Gainomax Recovery Vanilj (Norrmejerier Ek. För., Umeå, Sweden) poured into 2 glasses, and 2 bars (75 kcal/bar) of a Wasa Sandwich Cream Cheese Naturell (Barilla Sverige AB, Stockholm, Sweden), providing 650 kcal. This preload was consumed in its entirety within 10 min. Following the preload, participants again reported appetite ratings (08:30 h), and conducted the sweet taste intensity rating task (09:00 h).

### Sweet taste intensity

A standard food (yogurt; Mild Lättyoghurt Naturell, Arla Foods, Viby, Sweden) was presented to which sucrose was added at different concentrations: 2 %, 5 %, 9 %, 15 %, and 22 % and 29 % (w/w; i.e., gram sucrose per 100 ml yoghurt). Participants tasted a single spoonful and rated sweetness intensity and hedonic value (pleasantness) of the different sucrose concentrations on a 100-mm VAS, anchored with the terms “not at all” and “extremely”. Participants were then asked to return the remaining yogurt and to neutralize their taste by eating plain crackers and drinking water. This procedure was repeated for all six sucrose concentrations. The order of presentation was randomized within participants.

### Biochemical analysis

Blood samples were centrifuged immediately after sampling. The supernatant was stored at −80 °C, for analysis of plasma ghrelin and glucose. Concentrations of total ghrelin were assessed using commercially available ELISA kits for humans (EZGRT-89 K; Millipore, Billerica, MA, USA). Plasma glucose was measured using routine assays (hexokinase method, Aeroset; Abbott Diagnostics, North Chicago, IL, USA).

### Data analysis

Data were analyzed using SAS (version 9.5; SAS Institute Inc.), and are presented as means (± SEM) unless otherwise indicated. The effect of sleep deprivation on ratings of sweetness intensity and pleasantness was tested using repeated ANOVA measures (within-subject factors: Sleep/TSD, Sucrose concentration). In order to check that intra-individual differences did not account for nonsignificant effects in the small sample size, we repeated these analyses using normalized values. Since TSD was hypothesized to increase plasma ghrelin and feelings of hunger, a one-tailed p value of < 0.05 was considered significant. In case of the sweet perception task, a two-tailed p value < 0.05 was considered significant.

## Results

### Appetite sensations

Hunger ratings on the adaptation day did not differ between the sleep and TSD conditions (data not shown). In the morning after nocturnal wakefulness, participants reported greater hunger than they did after normal sleep (TSD vs. Sleep, 76 ± 7 vs. 61 ± 6 mm, p = 0.01 at 07:00 h; 80 ± 4 vs. 69 ± 6 mm, p = 0.08 at 08:00 h; 43 ± 7 vs. 33 ± 7 mm, p = 0.04 at 08:30 h [i.e., 20 min after the preload]). However, no significant differences between the TSD and sleep conditions were observed when the change in hunger before (i.e., 08:00 h) and after the preload (i.e., 08:30 h) was analyzed (TSD vs. Sleep, −37 ± 6 vs. −36 ± 5 mm). Other appetite ratings (fullness, desire to eat, and prospective consumption) yielded similar results, with corresponding changes following the preload (data not shown).

### Sweet taste perception and pleasantness

**Fig. 1:**
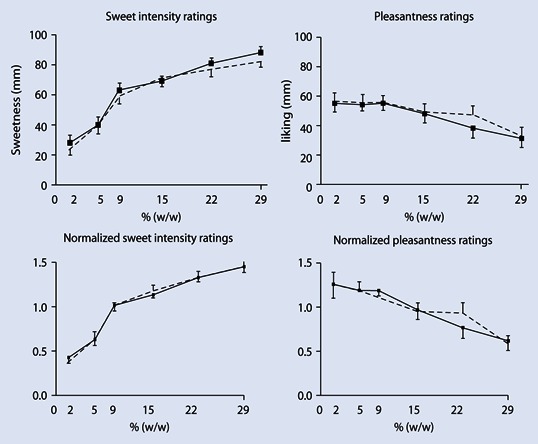
Mean (± SEM) absolute and normalized ratings of sweet taste intensity and pleasantness of yogurts with increasing sucrose concentrations: 2 %, 5 %, 9 %, 15 %, 22 %, and 29 % (i.e., gram sucrose per 100 ml yoghurt, % [w/w]) after both sleep (*dashed line*) and total sleep deprivation (*solid line*) conditions. As indicated by repeated measures ANOVA, ratings of sweet intensity or pleasantness of the yogurt samples with varying sucrose concentrations did not significantly (i.e., p < 0.05) differ between conditions

Ratings of sweet intensity or pleasantness of the yogurt samples with varying sucrose concentrations did not differ between conditions (Fig. 1). Similar null results were obtained when ratings of the sweet taste perception task were normalized (Fig. 1).

### Sleep recordings

Sleep in the sleep condition was typical for laboratory conditions (Tab. 1). The sleep-onset latency was 8 ± 4 min, and sleep efficiency was 92 ± 1 %.

**Tab. 1:** Polysomnographic characteristics (mean ± SEM) of the second night in the sleep condition

Parameter	
Time in bed (min)	480
Sleep onset latency	8 ± 4
After sleep onset	
Total sleep time (min)	442 ± 6
Wake (min; B)	30 ± 3
Sleep stage 1 (min)	5 ± 1
Sleep stage 2 (min)	219 ± 11
Slow wave sleep (min)	115 ± 6
Rapid eye movement (REM) sleep (min)	103 ± 8
Sleep efficiency (%)^a^	92 ± 1

^a^Sleep efficiency = total sleep time/time in bed.

### Glucose and hormonal measurements

Plasma glucose measurements did not differ between the TSD and sleep conditions (TSD vs Sleep, 5.4 ± 0.5 vs. 5.3 ± 0.4 mmol/l). Plasma concentrations of total ghrelin were higher after TSD than after a night of sleep (TSD vs. Sleep, 442 ± 61 vs. 390 ± 44 pg/ml, p = 0.04).

## Discussion

Here we show that one night of wakefulness (which is common in shift workers) increases morning plasma concentrations of the hunger-promoting hormone ghrelin in healthy young men. In contrast, perception of sweet taste tested in a broad range of sucrose concentrations was not affected. This suggests that altered processing of sweet tastes is an unlikely the mechanism by which one night of total sleep deprivation (TSD) affects the homeostatic and hedonic control of eating in humans [6, 14, 15, 16, 17].

It has been demonstrated that humans who receive an intravenous bolus of the hormone ghrelin while viewing images of palatable food show an increased activity in brain areas intimately linked to gustatory and reward processing (e.g., amygdala, orbitofrontal cortex, anterior insula, and striatum) [18]. In line with these observations, high circulating levels of ghrelin have been linked to enhanced consumption and preference for sweet tasting food in animals [9]. Against this background, we hypothesized that acute sleep deprivation—a condition that causes an increase in morning plasma concentrations of ghrelin, as shown here and by others [5, 19]—would modulate the perception of sweet taste in healthy young men. Such a finding would offer a potential mechanism through which sleep loss may promote overeating in humans. However, in contrast to our hypothesis, a night of wakefulness affected neither taste intensity nor pleasantness ratings when subjects were administered six ascending concentrations of sucrose in yogurt samples. There are a variety of explanations for why we did not observe the predicted effects. A range of metabolic, physiological, and genetic variables that vary from person to person are known to moderate the ability to perceive sweetness [20]. With this in mind, we cannot rule out the possibility that the potential effects of TSD on sweet perception were masked by inter-individual differences in sweet taste perception resulting from differences in these variables [21]. However, normalizing the results of the sweet taste perception task to account for the ability to perceive sweetness failed to produce significance. Another possibility is that the caloric preload reduced the sensitivity of the test, and that participants in the fasted state would have shown significant differences between conditions. Additionally, caution should be exerted when extrapolating our results, as the experiment focused exclusively on sweetness, at the exclusion of other potentially significant tastes such as salt, bitter, sour, and umami. Sleep loss has been shown to increase the responsively to stress in humans [22]. As stress can cause a generalized decrease in sensitivity to rewards (i.e., anhedonia) [23], our negative results do not exclude that acute sleep deprivation in conjunction with stress may alter the way humans perceive sweet taste. Finally, whether sweet taste perception remains also stable under periods of chronic partial sleep deprivation, or whether our results generalize to females or other age groups, requires further investigation.

## Conclusion

Our data add further evidence that acute sleep loss increases circulating concentrations of the hunger-promoting hormone ghrelin in healthy young men [4, 5]. In contrast, TSD did not produce differences in sweet taste perception. This suggests that the increase in daytime food intake after nocturnal sleep deprivation may be caused by endocrine mechanisms independently from the subjective perception of sweetness.

## References

[1] Patel (2008). Obesity.

[2] Schmid (2009). Am J Clin Nutr.

[3] Spiegel (2004). Ann Intern Med.

[4] Taheri (2004). PLoS Med.

[5] Benedict (2011). Am J Clin Nutr.

[6] Benedict (2012). J Clin Endocrinol Metab.

[7] Reynolds (2012). PLoS One.

[8] Nakazato (2001). Nature.

[9] Disse (2010). Physiol Behav.

[10] Shin (2010). PLoS One.

[11] Egecioglu (2010). Addict Biol.

[12] Skibicka (2012). Addict Biol.

[13] Rechtschafen (1968). Los.

[14] St-Onge (2011). Am J Clin Nutr.

[15] Brondel (2010). Am J Clin Nutr.

[16] Nedeltcheva (2009). Am J Clin Nutr.

[17] Bosy-Westphal (2008). Obes Facts.

[18] Malik (2008). Cell Metab.

[19] Schmid (2008). J Sleep Res.

[20] Drewnowski (1997). Annu Rev Nutr.

[21] Looy (1991). Chem Senses.

[22] Meerlo (2008). Sleep Med Rev.

[23] Papp (1991). Psychopharmacology.

